# Supplementary figures and images for: Signs and Symptoms of Acute Bowel Inflammation and the Risk of Progression to Inflammatory Bowel Disease: A Retrospective Analysis
Source: J Clin Med. 2022 Aug 6;11(15):4595. doi: 10.3390/jcm11154595 (PMC9369956; doi:10.3390/jcm11154595)

Time to IBD

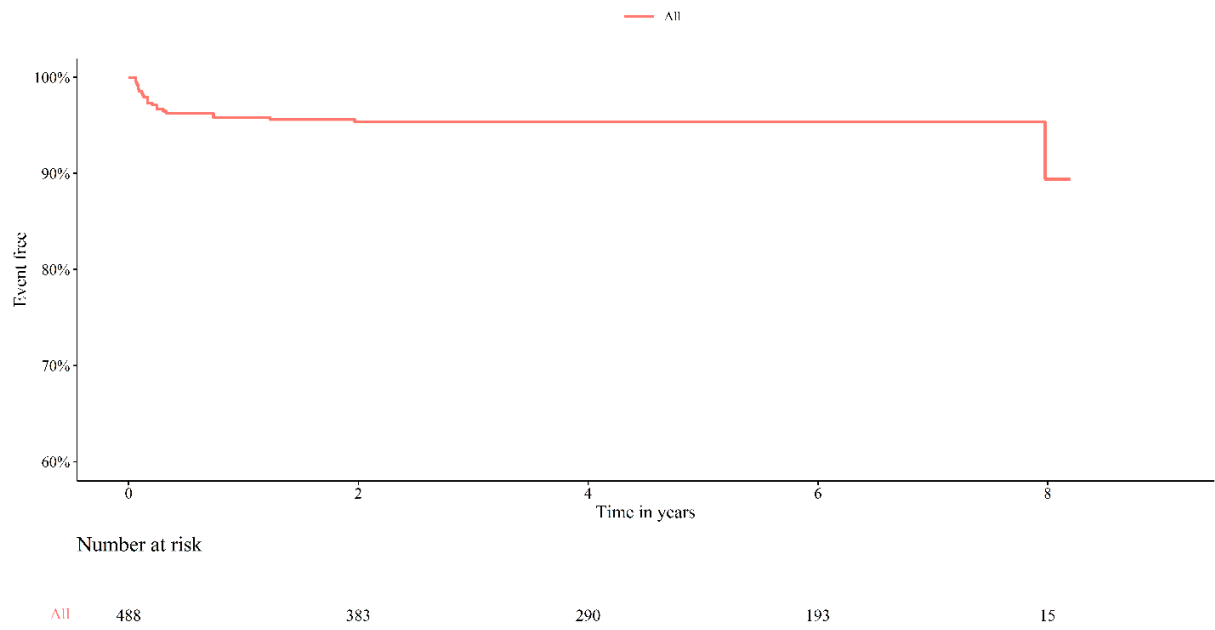

Supplement: Supplementary file 1 [file jcm-11-04595-s001.zip › jcm-1813170-supplementary.pdf]
